# Monitoring hospital hygiene by luminescence methodology

**DOI:** 10.1186/2047-2994-4-S1-P30

**Published:** 2015-06-16

**Authors:** F Vieira, I Neves, I Devesa, D Peres

**Affiliations:** 1 Infection Control and Antimicrobial Resistance Unit, Unidade Local de Saúde de Matosinhos, Matosinhos, Portugal; 2 Infectious Diseases Unit, Unidade Local de Saúde de Matosinhos, Matosinhos, Portugal

## Introduction

The environment hygiene (EH) is a basic measure in the prevention of healthcare associated infection. Until recently there was no methodologies that evidenced the cleanness of the environment, but now published studies demonstrated how to turn EH a science based on evidence.

## Objectives

Monitor the effectiveness of the hygiene cleaning procedures in a hospital environment, using a bioluminescence methodology.

## Methods

Acute care hospital with implemented antiseptics/disinfectants policy and specific hygiene cleaning plans in every unit. Evaluation of the effectiveness of the EH through bioluminescence methodology using the “Kikkoman Lumitester PD-20®”. In this system a surface sample is collected and, after inserted in the machine, the organic residues are measured through quantification of adenosine triphosphate (ATP), which is proportional to the light intensity generated. Healthcare units with higher risk have been chosen to be studied (intensive care units, operating rooms, radiology and medicine department). In these settings, 10 samples were collected, in different structures, before cleaning and other 10 after cleaning and process supervision by the head nurse.

## Results

Before the EH, 30% of samples were considered “dirty”, 50% “clean” and 20% “intermediate”. After cleaning 20% were “dirty”, 70% “clean” and 10% “intermediate”. The results obtained were not homogeneous in the different studied units, however some showed a significant reduction after cleaning and others evidenced errors in the procedures.

## Conclusion

This method allowed evidencing the EH and seems to support that validation by traditional methods (visual audits) is not enough. Since it’s a quantitative method, it allows to inform professionals of errors in the cleaning process and promotes continuous improvement.

## Disclosure of interest

None declared.

